# Enhanced Extraction of Bioactive Compounds from Red Grape Pomace: Optimizing Ultrasound-Assisted Extraction with Ethanol and NaDES as Solvents

**DOI:** 10.3390/antiox14050526

**Published:** 2025-04-27

**Authors:** Nicoleta Balan, Silviu Măntăilă, Gabriela Râpeanu, Nicoleta Stănciuc

**Affiliations:** Faculty of Food Science and Engineering, “Dunarea de Jos” University of Galați, 800008 Galați, Romania; nicoleta.balan@ugal.ro (N.B.); silviu.mantaila@ugal.ro (S.M.); gabriela.rapeanu@ugal.ro (G.R.)

**Keywords:** bioactive, grape pomace, ultrasound extraction, NaDES, bioaccesibility

## Abstract

This study aims to investigate two types of solvents, ethanol and natural deep eutectic solvent (NaDES), using the ultrasound-assisted extraction techniques, in order to analyze their efficiency and ability to extract polyphenolic compounds from red grape pomace. The optimization and validation of the most feasible extraction conditions leading to maximization of the dependent variables (total anthocyanins, polyphenols, flavonoids and antioxidant activity), were carried out using response surface methodology with a central composite design. For ethanol extraction, the validated optimal conditions were at 35 °C for 22.5 min and a concentration of 70% ethanol. The values obtained under these conditions were 105.32 mg cyanindin-3-glucoside (C3G)/g DW, 465.81 mg gallic acid equivalents (GAE)/100 g DW, 15.3 mg catechin equivalents (CE)/100 g DW and 1414.15 mMol Trolox/g DW, respectively. Concerning the extraction using NaDES, consisting of a 1:2:1 molar mixture of choline chloride, lactic acid and water, the optimal conditions that led to a profile consisting in 57.58 mg C3G/g DW, 414.04 mg GAE/100 g DW, 15.8 mg CE/100 g DW and 7.28 mMol Trolox/g DW, respectively, were at 60 °C for 60 min and a solvent volume of 10 mL. Two different chromatographic profiles were obtained, with 12 polyphenolic compounds identified in ethanolic extracts and only 5 in NaDES, respectively. The in vitro digestion study revealed the high bioaccessibility of polyphenols in the gastric environment, with a drastic decrease in simulated intestinal fluid. The results are valuable in terms of identifying the best extraction conditions for polyphenols using alternative, non-toxic, ecofriendly solvents.

## 1. Introduction

In general, the agricultural and industry produces increasing amount of waste and by-products, which can cause serious environmental contamination problems if not managed immediately, because they do not decompose quickly in the environment. It has been suggested that the global annual production of grape is about 75 million metric tons, accounting for 8–11 percent of total fruits produced in the world [1]. The industrial processing of grape in juice and wine lead to a high percentage of grape pomace, which accounts for between 10 and 30% of the total weight of the grapes and is composed of skins, seeds, pedicels and other organic solids resulting from the technological process [2]. FAO [3] suggested that the production of 6 L of wine generates one kg of grape pomace, which amounts to 10.5–13.1 million tons of grape pomace being produced in the world annually.

During winemaking, the low extraction process allows retaining the following in pomace: large amounts of condensed tannins, mainly proanthocyanidins composed of procyanidin and prodelphinidin units, anthocyanins, catechins, flavanols, alcohols and stilbenes, as well as benzoic and p-coumaric acids [1]. Llobera and Cañellas [4] suggested that red grape pomace (RGP) may reach a value of about 50 g/kg dry matter in condensed tannins and total polyphenols. However, grape pomace, in general, contains as main compounds polysaccharides (30%), and some other compounds such as acid pectic substances, lignin and proteins, in lesser percentages [5].

Polyphenol compounds are plant secondary metabolites chemically characterized by an aromatic ring containing one or more hydroxyl substituents. These compounds exert various effects that contribute to improving health and reducing the risks associated with certain diseases. They exhibit a wide range of biological properties, including antiallergenic, antimicrobial, anti-inflammatory, antiaging, antitumor, antioxidant, antilipotropic, antithrombotic, cardioprotective, insulinotropic and vasodilator effects [6]. The two main classes of polyphenolic compounds in grape pomace are flavonoids (anthocyanins, flavonols, flavan-3-ols) and non-flavonoids (phenolic acids and stilbenes). These compounds have attracted significant attention due to their numerous bioactive properties that are beneficial to human health, including the prevention of chronic diseases, cardiovascular diseases, cancer and diabetes. Additionally, their antimicrobial activity and antioxidant capacity make them valuable for potential biotechnological applications [7,8]. Additionally, through their pro-oxidative effect, polyphenols can induce apoptosis of cancer cells, while by inhibiting the angiogenesis process, these compounds contribute to reducing tumor growth. At the same time, polyphenols diminish the metastatic potential by decreasing the adhesiveness and invasiveness of malignant cells [9].

Current studies have demonstrated that supplementation with grape polyphenols can reduce plasma glucose concentration in both animals and human subjects. In addition, prolonged supplementation with these compounds has been associated with an improvement in glucose metabolism in people diagnosed with metabolic syndrome and type 2 diabetes [10].

Grape pomace extractable components may be used in different applications, such as food, feed, ingredients of functional foods, cosmetics and nutraceuticals; and to replace the synthetic dyes and preservatives in foods [11]. However, when considering the use of polyphenolic compounds in different applications, several critical issues should be considered, namely the extraction method and parameters, the bioactive low bioaccesibility and bioavailability and their low stability during storage. The identification of efficient extraction methods aiming to increase the yield of phenolic compounds while maintaining their stability in the extract, in a sustainable and environmentally friendly manner, is a current trend in scientific research [12,13,14].

One of the most commonly used green techniques for recovering phenolic compounds from by-products which is widely addressed in research studies is ultrasound-assisted extraction (UAE). A study conducted by Drosou et al. [15] suggested that UAE, using a 1:1 water–ethanol ratio as a solvent, resulted in the highest phenolic compound yield from both untreated and dried grape pomace, compared to microwave-assisted extraction and Soxhlet extraction. Liu et al. [16] highlighted that UAE, compared to traditional methods, improves the extraction efficiency of flavonoids, anthocyanins and the antioxidant activity of grape pomace extract. Additionally, UAE offers advantages such as simplicity, reduced extraction time, lower environmental impact due to reduced energy consumption and the possibility of solvent recycling. However, new approaches for efficient extraction of polyphenols from different matrices are studied, using eco-friendly solvents, such as natural deep eutectic solvents (NaDES). NaDESs are promising alternative to conventional extraction solvents, offering important advantages such as biodegradability, low toxicity and the ability to extract a wide range of compounds from different matrices. In principle, NaDESs are mixtures of two or more compounds in a certain molar ratio, a hydrogen donor and a hydrogen acceptor, respectively, which through molecular interactions undergo a decrease in melting point, becoming liquid at room temperature [17].

Hence, the main objective of this study was to use optimize and validate the ultrasound-assisted extraction of polyphenols from RGP using two types of solvents. In this purpose, the response surface methodology (RSM) through central composite design (CCD) was used for the optimization and validation of extraction parameters. The two solvents were a hydroalcoholic solution and choline chloride–lactic acid–water solution, with a molar ratio 1:2:1. The validated extracts were further tested for in vitro digestibility in order to evidence the stability of extracted polyphenols in the gastrointestinal environment.

## 2. Materials and Methods

### 2.1. Chemical and Reagents

Potassium chloride, sodium acetate, aluminum chloride, Folin–Ciocalteu reagent, 2,2-diphenyl-1-picrylhydrazyl (DPPH), formic acid ≥ 98%, choline chloride ≥ 98% ((2-hydroxyethyl)trimethylammonium chloride), lactic acid 90% and Trizma were purchased from Sigma-Aldrich (Millipore Sigma, Steinheim, Germany). Ethanol > 96% was purchased from S.C. Maraton ·92 Impex S.R.L. (Bucharest, Romania), and methanol for HPLC, ≥99.9%, was purchased from Honeywell (Seelze, Germany). Monosodic and dibasic sodium were purchased from Chimreactiv S.R.L. (Bucharest, Romania), and sodium carbonate was purchased from SC. Chemical Company SA (Iasi, Romania). Pepsin from porcine gastric mucosa and pancreatin from porcine pancreas were purchased from Sigma-Aldrich (St. Louis, MI, USA). The standard compounds used for chromatographic analysis were purchased from Sigma-Aldrich (Millipore Sigma, Steinheim, Germany). All the other reagents were of analytical grade.

### 2.2. Plant Material

The unfermented red grape pomace (RGP) (*Vitis vinifera* L.) from the Fetească Neagră and Merlot varieties was sourced from the Bratu winery, located in Odaia Manolache village, Vânători commune, Galați County (45°33′27.5182″ N, 28°0′21.7552″ E, Romania). RGP was stored at −20 °C in refrigerated containers until use. RGP drying was performed by hot air convection (Stericell 111 ECO Hot AIR Sterilizer; MMM Medcenter Einrichitungen GmbH, München, Germany) at 35 °C until a moisture content of 8% was reached. Subsequently, the dried material was grounded using an electric grinder (Coffee grinder Heinner HCG-150SS; Heinner, Network One Distribution, Bucharest, Romania) and stored in vacuum-sealed bags under refrigeration conditions (4 °C).

### 2.3. Ultrasound-Assisted Extraction (UAE)

#### 2.3.1. Hydroalcoholic Solutions

Extraction of polyphenolic compounds from dried RGP was performed using an ultrasonic bath (Digital Ultrasonic Bath Mod. DU-32; 131 ARGOLAB; Capri, Italy). Over the weighed samples (1 g) in vials, 9 mL of the solvent with different ethanol/water ratios (*v*/*v*, 50–90%) was added and subjected to ultrasound at different temperatures (25–45 °C) and times (15–30 min). The ultrasound parameters were kept constant at a frequency of 40 kHz and power of 100 W. For maintaining the temperature constant, cold water was added in the ultrasonic bath. Further, the hydroalcoholic extract was centrifuged at 6000× *g* for 10 min and at temperature of 4 °C. The supernatant was collected in another vial and stored in the dark at 4 °C until future analyses.

#### 2.3.2. Natural Deep Eutectic Solvents (NaDES)

The NaDES used for the extraction of polyphenolic compounds from unfermented RGP is choline chloride, lactic acid and water in a molar ratio of 1:2:1. According to Osamede Airouyuwa et al. [12], this molar ratio significantly enhances the extraction of anthocyanins from plant material compared to other NaDES, due to the polar–polar interaction created and the monoprotic nature of lactic acid. The addition of water reduces the viscosity of the solution, thereby improving mass transfer from the plant material, respectively, increasing the extraction yield of anthocyanins [18].

The independent variables applied in the NaDES-based extraction process include solid–liquid ratio, extraction time and temperature, following the same procedural steps for obtaining the supernatant: ultrasonication, centrifugation, collection and storage until future analyses, as described above.

### 2.4. Experimental Design

For the optimization and validation of extraction using ethanol and NaDES, the response surface method (RSM) was used to analyze the effects of independent variables on the responses: total anthocyanin content (TAC), total polyphenol content (TPC), total flavonoid content (TFC) and antioxidant activity (DPPH) of the obtained extracts. Additionally, RSM was used to identify the optimal parameters for maximizing the extraction yield of polyphenolic compounds from RGP.

The three independent variables used in the RSM for optimizing and validating the extraction were as follows: extraction temperature (A), which ranged from 25 to 45 °C; extraction time (B) in the ultrasonic bath, which ranged from 15 to 30 min; and ethanol concentration (C), which varied between 50 and 90%, *v*/*v*. These factor ranges were established according on the scientific literature to enhance the extraction of polyphenolic compounds while protecting them from degradation [19,20].

The independent variables introduced in the CCD were temperature (A), ranging from 40 to 60 °C; extraction time (B), varying between 30 and 60 min; and the solid–liquid (S/L) ratio of NaDES (C) used, which varied from 1:10 to 1:20 mL. The temperature and extraction time of the compounds were adapted according to the study carried out by Li et al. [21], which focused on the identification of the best NaDES that would lead to obtaining a stable extract with an increased yield of anthocyanins from grape pomace.

### 2.5. Total Anthocyanin Content (TAC)

The total anthocyanin content of RGP samples was determined by the pH differential method, as described by Liu et al. [16], with some methodological adjustments. Thus, 200 µL of the samples to be analyzed were added to cuvettes, to which 800 µL of buffer solution with pH = 1.0 (KCl) was added. Similarly, 800 µL of buffer solution with pH = 4.5 was added to other cuvettes. The absorbance value was measured, in triplicate, independently, at wavelengths of 510 nm and 700 nm, using a spectrophotometer (Biochrom; Libra 22 UV/Visible Spectrophotometer; 177 Holliston, MA, USA), after the samples had been kept in the dark for 15 min at room temperature. The absorbance calculation was performed according to Equation (1), whereas the TAC was calculated based on Equation (2) and expressed in mg cyanidin-3-*O*-glucoside equivalents/grams dry weight (mg C3G/g DW):

(1) $$Abs={\left(A_{510}-A_{700}\right)}_{pH\;1.0}-{\left(A_{510}-A_{700}\right)}_{pH\;4.5}$$

(2) $$TAC=\frac{Abs}{e\times L}\times MW\times D\times \frac{V}{G}$$

where Abs—absorbance calculated based on Equation (1), e—molar absorbance of cyanidin-3-*O*-glucoside (26,900), L—length of the cuvette (1 cm); MW—molecular weight of cyanidin-3-*O*-glucoside (449.2 g/Mol); D—dilution factor; V—final volume (mL); G—weight of dry pomace (g).

### 2.6. Total Polyphenol Content (TPC)

The Folin–Ciocalteu colorimetric technique, as described by Gutfinger [22], with subsequent modifications introduced by Serea et al. [23], was applied to determine the total phenolic content in RGP extracts. The absorbance at a wavelength of 765 nm was measured using a spectrophotometer (Biochrom; Libra 22 UV/Visible Spectrophotometer; 177 Holliston, MA, USA). Readings were made in triplicate for each sample, and the results obtained were expressed in milligrams of gallic acid equivalents per 100 g of DW (mg GAE/100 g DW).

### 2.7. Total Flavonoid Content (TFC)

The total flavonoid content of RGP extracts was determined using an AlCl3-based colorimetric assay adapted from the procedures described by Yang et al. [24] and Shi et al. [25]. A total of 250 µL of the sample was added to the cuvettes, followed by 250 µL of 2% AlCl_3_ solution and 1500 µL HPLC-purified methanol. The samples were incubated in the dark for 15 min at room temperature. The absorbance at 440 nm was measured using a spectrophotometer (Biochrom; Libra 22 UV/Visible Spectrophotometer; 177 Holliston, MA, USA), in triplicate. The results were expressed in milligrams of catechin equivalents per gram DW (mg CE/100 g DW).

### 2.8. Antioxidant Activity

According to the studies conducted by Serea et al. [23], the 2,2-diphenyl-1-picrylhydrazyl (DPPH) method was applied to evaluate the antiradical activity of the extracts. The optical absorption of the samples was measured with (Biochrom; Libra 22 UV/Visible Spectrophotometer; 177 Holliston, MA, USA), at a wavelength of 515 nm. To establish a baseline, a control sample was prepared by adding methanol and was analyzed simultaneously with the test samples, and all results were obtained in triplicate. The results obtained were expressed in mMol Trolox equivalents/g DW.

### 2.9. High-Performance Liquid Chromatography

The identification of polyphenolic compounds was performed at a wavelengths of 280 nm, 320 nm and 520 nm according to the method described by (Mërtiri et al. [26] using an Agilent 1200 HPLC equipment (Agilent Technologies, Santa Clara, CA, USA), which is equipped with a degasser, quaternary pumping system, column and auto-wave detector. The obtained data were automatically processed by the Agilent ChemStation software, version Rev. B.04.03, based on existing calibration curves with standards compounds. The results were obtained in triplicate. The separation of the compounds was performed using the BDS Hypersil C18 column (150 × 4.6 mm, 5 μ), the column temperature was set at 30 °C, the injection volume was 10 μL and the flow rate was 1 mL/min. The 100% methanol (solvent A) and 10% formic acid (solvent B) represented the mobile phase, and the gradient progression was as follows: 0–20 min. 9% A, 91% B, 20–30 min. 35% A, 65% B, 30–40 min. 50% A, 50% B, 40–45 min. 9% A, 91% B. The results were expressed in mg/mL.

### 2.10. In Vitro Digestibility of Grape Pomace Extracts

The digestibility of the two extracts obtained was performed according to the method described by Stoica et al. [27]. The protocol is based on the use of a statistic model simulating the gastric and intestinal human digestion of the extracts obtained. The extracts used in digestion were obtained under optimal extraction conditions, by repeating the experiments three times, in order to exhaust the pomace in the bioactive. In a ratio of 1:1, optimized extract (ethanolic, respectively, NaDES) was mixed with a Tris-HCl buffer solution (10 mM, pH = 7.7). Simulated gastric digestion was performed by mixing 5 mL of the initial mixture and 10 mL simulated gastric fluid (SGF) containing porcine pepsin (40 mg/mL in 0.1 M HCI, pH = 3.0). After two hours of digestion, 5 mL of the digestate was mixed with simulated intestinal fluid (SIF) containing pancreatin from porcine pancreas (2 mg/mL in 0.9 M sodium bicarbonate, pH = 7.0). The samples were maintained in a shaker (Medline Scientific, Chalgrove, Oxon, UK) at 150 rpm and 37 °C for 4 h. Before analysis, the samples were centrifuged at 14,000 rpm for 10 min at 4 °C. The analyses were performed by sampling every 30 min of one milliliter of the digestate, and TAC, TPC, TFC and DPPH were determined.

The results are discussed in terms of bioaccessibility, calculated on the basis of Equations (3) and (4) [28]:

Gastric bioaccessibility (%) = (gastric fraction/bioactive content) × 100(3)

Intestinal bioaccessibility (%) = (gastric fraction/total phenolic content) × 100(4)

### 2.11. Statistical Analysis

Experimental design, analysis, optimization and validation of the results were performed using Design-Expert software version 13.0 (Stat-Ease, Minneapolis, MN, USA) and Microsoft^®^ Excel^®^ for Microsoft 365 MSO version 2412 build 16.0.18324.20240 (Microsoft, Redmond, WA, USA) for the calculation of dependent variables. The ANOVA for quadratic model was applied to determine the significance of the model, and the independent variables in the second-order polynomial equation generated by the software, with a significance level (α) of 0.05. By evaluating the multiple coefficient of determination (R^2^), the adjusted correlation coefficient (Adj-R^2^) and the predicted correlation coefficient (Pred-R^2^), respectively, the significance of lack of fit and the quality of the model can be evaluated.

## 3. Results and Discussion

### 3.1. Optimization of UAE Process Parameters

The first part of this study aimed to identify the optimal conditions for the extraction of polyphenolic compounds from RGP using CCD, independent variables (temperature, extraction time, ethanol concentration, %) for the solid–liquid conventional extractions (ethanol/water, *v*/*v*) and independent variables (temperature, extraction time and solid–liquid ratio) for the NaDES. The responses measured generated by the RSM were the following: TAC, TFC, TPC and antioxidant activity.

In Table 1 and Table 2 are presented the matrices modeled by CCD for the two solvents used.

Table 3 shows the statistical parameters calculated using ANOVA for the two experimental designs generated by CCD.

Thus, the experimental results obtained were fitted on a quadratic polynomial model, in which the coefficient values of R^2^ > 0.99, the significance of *p*-value for lack of fit is >0.05, these indicating the quality and the adequacy of the two experimental designs.

### 3.2. Influence of Independent Variables on TAC

As can be seen in Table 1, for ethanol that was used as solvent, the TAC ranged from 45.77 ± 1.09 to 106.21 ± 2.50 mg C3G/g DW depending on the variation in extraction time, temperature and ethanol content in the solvent for each run. According to the ANOVA findings, the quadratic model for TAC, the terms having significance due to *p*-value less than 0.05 are as follows: A (<0.0001), B (<0.0001), C (<0.0001), AC (<0.0001), BC (0.0003), A^2^ (0.0002), B^2^ (<0.0001) and C^2^ (<0.0001), while AB (0.1430) has no significance.

TAC = 105.91 − 2.05A − 2.98B + 4.33C + 0.3162AB − 1.19AC + 3.22BC − 15.63A^2^ − 14.67B^2^ − 18.57C^2^ (5)

According to the coefficients in the regression (Equation (5)), the ethanol concentration (B) has a positive impact on anthocyanin extraction and its interaction with extraction time (C). In contrast, the interaction between temperature and solvent showed negative influences of TAC extraction from RGP. Similar results were presented in the study carried out on the grapes skin of the Băbească Neagră variety by Serea et al. [23]. They showed that the solvent significantly influences the extraction of TAC and emphasized the negative impact of the interaction between temperature and extraction time, quadratic time and temperature, following the optimization of the extraction using CCD.

**Figure 1:**
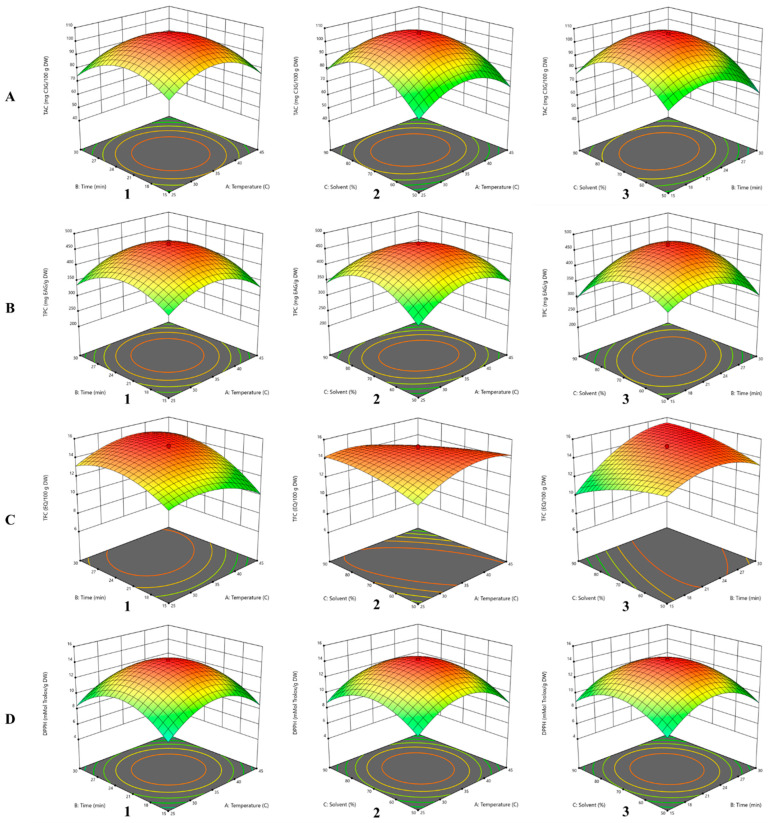
Three-dimensional response surface and two-dimensional contour plots for the ethanol extract, analyzing the interaction effect between independent variables on the TAC ((**A**)—(**1**): temperature–time; (**2**): temperature–solvent; (**3**): solvent–time), TPC ((**B**)—(**1**): temperature–time; (**2**): temperature–solvent; (**3**): solvent–time), TFC (**C**)—(**1**): temperature–time; (**2**): temperature–solvent; (**3**): solvent–time), DPPH ((**D**)—(**1**): temperature–time; (**2**): temperature–solvent; (**3**): solvent–time). Green indicates the lowest response values, and as the values increase, the color changes, with deep red representing the highest response values obtained.

In Figure 1(A2) it can be observed that at low temperature (18.18 °C) and low ethanol concentration (36.36%), the extraction of TAC is not facilitated. Thus, ethanol plays an important role together with ultrasound treatment in the extraction of anthocyanins from the red grape skin cell wall, while applying a higher temperature (51.82 °C) and a solvent with a higher ethanol concentration (70%), negatively influencing the extracted amount of these compounds due to the reduced stability at higher thermal treatments [19,29].

Analyzing Figure 1(A1–A3), the interaction effects of independent variables, temperature–time, temperature–ethanol concentration and time–ethanol concentration, on TAC extraction were revealed. Figure 1(A3) shows that the interaction between extraction time and ethanol concentration has the highest impact on TAC extraction, the maximum values being obtained at 70% ethanol and 22.5 min (106.21 ± 2.50 mg C3G/g DW). Zhou and Yang. [30] obtained a significantly lower anthocyanins content in relative similar extraction conditions, 78.9% ethanol with pH 7.0, temperature of 63.8 °C for 48 min, by using a S/L ratio of 1:15 ratio (*w*/*v*), resulting in a yield of 193.547 mg/100 g anthocyanins from GP.

The significant terms in the model in the case of TAC extraction from RGP with NaDES are A (<0.0001), B (<0.0001), C (<0.0001), AB (<0.0001), BC (<0.0001), A^2^ (0.0003), B^2^ (0.0002) and C^2^ (<0.0001), while AC has no significance due to its *p*-value (0.4125), which is higher than the confidence interval (95%). Table 2 shows the values obtained for TAC for the generated experiments; thus, the anthocyanin content ranged from 22.07 ± 0.89 to 56.84 ± 1.52 mg C3G/g DW.

TAC = 28.58 + 2.47A + 4.49B − 8.99C + 3.00AB − 0.21AC − 1.34BC + 1.05A^2^ + 3.75B^2^ +3.26C^2^ (6)

The coefficients in the generated second order polynomial Equation (6) for TAC indicated a high positive effect for factors B, B^2^, C^2^, AB and A on the yield of anthocyanin extraction from RGP, while factors C, BC and AC influenced the TAC in the extract in a negative manner.

**Figure 2:**
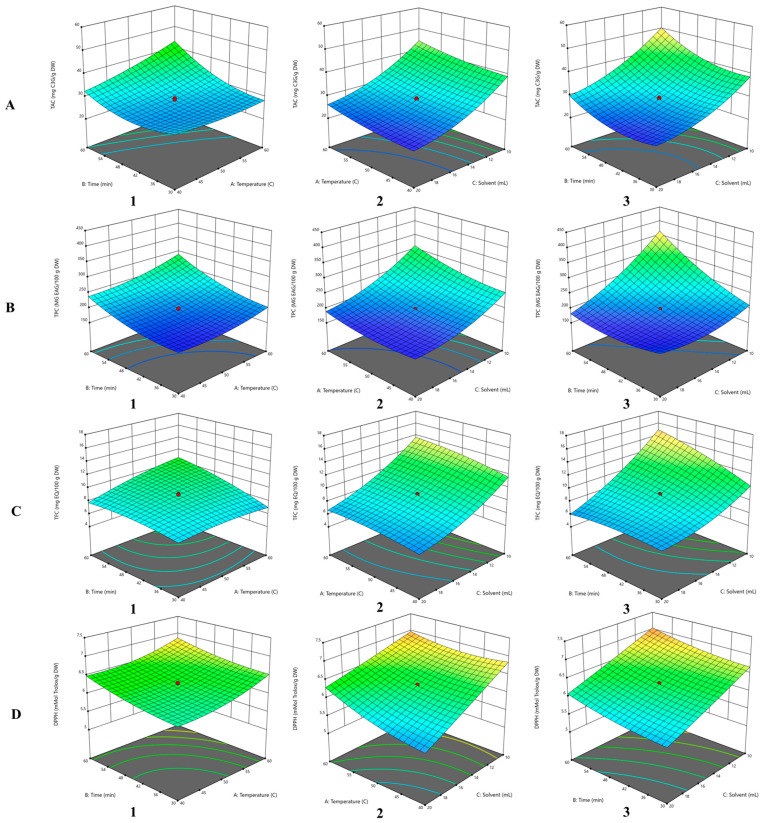
Three-dimensional response surface and two-dimensional contour plots for the NaDES extract, analyzing the interaction effect between independent variables on the TAC ((**A**)—(**1**) temperature–time; (**2**) temperature–solvent; (**3**) solvent–time), TPC ((**B**)—(**1**) temperature–time; (**2**) temperature-–solvent; (**3**) solvent–time), TFC (**C**)—(**1**) temperature–time; (**2**) temperature–solvent; (**3**) solvent–time), DPPH ((**D**)—(**1**) temperature–time; (**2**) temperature–solvent; (**3**) solvent–time). Deep blue indicates the lowest response value, and as the values increase, the color changes, with red representing the highest response value obtained.

By analyzing Figure 2(A1), it was established that there is a directly proportional correlation between the interaction of the factors temperature–extraction time (AB) and the response variable TAC in the case of NaDES extraction. Thus, at a temperature of 60 °C and an extraction time of 60 min, the highest amount of TAC was obtained from the extract. The optimal conditions for the extraction with the NaDES solvent applied in this study were Li et al. [21] in terms of temperature and extraction time of anthocyanins from grape pomace, where they obtained an extraction rate of 5.73 mg C3G/g DW. By analyzing Figure 2(A2,A3), the TAC extraction is directly influenced proportionally with increasing temperature and time, respectively, while the S/L ratio inversely influences the anthocyanin extraction from RGP.

### 3.3. Influence of Independent Variables on TPC

The TPC ranged from 209.48 ± 0.09 to 467.87 ± 1.41 mg GAE/100 g DW according to the experimental design for ethanol extraction, as shown in Table 1. Thus, in the case of the response variable TPC, B (0.0095), AB (0.0002), AC (<0.0001), BC (<0.0001), A^2^ (<0.0001), B^2^ (<0.0001), C^2^ (<0.0001) were those that showed model significance.

TPC = 461.88 + 1.15A + 4.49B − 0.51C + 10.56AB − 13.18AC + 31.20BC − 59.51A^2^ − 58.26B^2^ − 70.89C^2^ (7)

According to the regression Equation (7), the extraction time and temperature have a significant impact on the extraction of polyphenols. The interaction between extraction time (B) and ethanol concentration (C) showed the highest positive effect on the extraction yield of TPC, followed by the interaction between temperature and time, while the interaction between temperature (A) and solvent (C) negatively influences the extraction of polyphenols. The negative impact caused by the interaction between solvent (C) and temperature (A) can be explained by the fact that under either high or low thermal treatment and in the presence of high ethanol concentration, these compounds either undergo oxidation and thermal degradation processes or the conditions for efficient extraction are not met. As can be seen in Table 1, different values TPC were determined. Therefore, at a temperature of 18.18 °C and an ethanol concentration of 70%, an amount of 287.02 ± 0.97 mg GAE/100 g DW was obtained, whereas significantly lower values were found at temperature of 45 °C and 90% ethanol (209.48 ± 0.09 mg GAE/100 g DW). The three-dimensional surface and 3D graphical representations of the interaction of independent variables, temperature–time, solvent–temperature and solvent–time, are shown in Figure 1(B1–B3), which highlights their strong effects on the TPC extraction yield. Increasing ethanol concentration up to 70% concomitantly with increasing temperature up to 35 °C and time up to 22.5 min, respectively, leads to maximization of TPC following extraction with UAE.

The TPC values obtained by Abouelenein et al. [31] for the hydroalcoholic extracts obtained from three samples of pomace which originated from the red grape variety Lacrima di Morro d’Alba ranged from 41.23 to 48.30 mg GAE/g DW. The extracts were obtained with 70% ethanol acidified with HCl (0.01%) at a temperature of 25 °C, a frequency of 40 KHz and an ultrasound-assisted extraction duration of 60 min, followed by degreasing the supernatant with hexane.

Similar results being recorded by Muñoz et al. [32], who reported a TPC of 469 mg GAE/100 g DW for ethanol extraction from grape marc of the Carménère variety with the following optimal conditions: temperature 22 °C, amplitude 20%, extraction time 15 min and S/L ratio 1:3.2 (*w*/*w*). Nayak et al. [33] reported similar values for the extracts obtained from Cabernet Sauvignon pomace of 427.9 mg GAE/100 g for water extraction (solid–liquid ratio of 1:20 *w*/*v*) and 801.6 mg GAE/100 g for a hydroalcoholic extraction with 1:1 water–ethanol ratio for solvent (solid–liquid ratio of 1:20 *w*/*v*). Craciunescu et al. [34] recorded a TPC of 247.29 mg GAE/g DW in the Mamaia genotype; this genotype represents the hybrid resulting from the cross between Merlot, Babească Neagră and Muscat Ottonel. Arboleda Mejia et al. [35] obtained 260 mg GAE/100 g DW for the red grape marc mix (60% Cabernet Sauvignon, 30% Sangiovese and 10% Syrah), with an S/L ratio of 1:80 (*w*/*v*).

In the case of TPC, for all the independent variables, their interaction had a positive effect on this dependent variable (*p* < 0.0001). Similarly to the case of TAC, the S/L ratio, followed by the interaction of solvent volume and time, respectively, of the interaction of temperature had a negative effect on the extraction of polyphenolic compounds from the plant material (Equation (8)).

TPC = 196.73 + 19.48A + 37.28B − 51.57C + 6.95AB − 10.31AC − 40.36BC +12.79A^2^ + 17.63B^2^ + 21.89C^2^ (8)

The TPC in the case of NaDES solvent extraction ranged from 172.48 ± 3.55 to 414.70 ± 1.40 GAE/100 g DW. Similar results regarding the interaction between extraction time and temperature were reported by Gerçek et al. [36]. Following the application of the RSM using a Box–Behnken design with three levels and three factors, they observed that the maximum yield of TPC in chokeberry fruit was achieved at the highest temperature (50 °C) and extraction time (60 min), using a mixture of choline chloride and lactic acid at a molar ratio 1:2. These authors reported data in contrast with our study, evidencing a negative impact of temperature and extraction time on TPC, compared with the data presented in Equation (8), where temperature, time and their quadratics had a positive impact on the extraction of TPC. The positive effects of the independent variables temperature (A) and extraction time (B), their interaction (AB) and their quadratics (A^2^, B^2^) in the NaDES extraction of polyphenols were discussed previously in the literature [20], where the reduced solvent viscosity and surface tension improved the migration of the compounds from the plant material under the action of sonication. In combination with a prolonged extraction time, this phenomenon significantly improves the extraction yield.

The interaction between solvent volume and extraction time exhibited the greatest impact on TPC extraction from RGP in the case of extraction with NaDES, as can be seen in Figure 2(B3). As can be seen in the 2D contour plots, the TPC yield is influenced more by the S/L ratio used than by time and temperature, respectively, in the case of the interaction between these dependent variables.

Nguyen et al. [37] used the RSM with a CCD in order to optimize the extraction of TPC from grape pomace from the Red Cardinal variety using a DES composed of choline chloride and lactic acid for extraction. The highest TPC obtained in the study was 81.90 ± 1.06 mg GAE/g DW, a value obtained under the following experimental conditions: S/L ratio of 1:25, at a temperature of 70 °C for 118 min. In another study, Nguyen et al. [37] studied the impact of three different NaDES solvents in different molar ratios on the extraction of phenolic compounds, proanthocyanins and saponins from the grape pomace of Red Cardinal variety. They revealed that the solvent based on the molar ratio of 1:2 choline chloride and lactic acid, with an addition of 20% water at a temperature of 30 °C extraction time under shaking of 2 h and in a S/L ratio of 1/25 (*w*/*w*), resulted in the highest TPC yield value, of 42.53 ± 0.52 mg GAE/g DW.

Frontini et al. [38] reported lower values for the extraction of bioactive compounds from Negroamaro red grape pomace when using choline chloride in a 1:1 ratio, with tartaric acid, glycerol and lactic acid and water, reporting the lowest value for the extraction yield when mixing choline chloride and glycerol. Huamán-Castilla et al. [39] obtained higher values for TPC, varying from 33.39 to 49.22 mg GAE/g dried pomace (DP) when using DES at atmospheric extraction, and of 51.62 to 62.44 mg GAE/g DP when using hot pressurized liquid extraction.

The significant variations in bioactive compounds are given by the nature and composition of the solvent, but especially by its polarity, with polyphenols being generally polar compounds. The presence of water in the solvent composition increases the extraction yield and solubilization of polyphenols due to the weakening of hydrogen bonds established with proteins [40].

### 3.4. Influence of Independent Variables on TFC

The *p*-value indicates that all the terms A (0.0432), B (<0.0001), C (0.0076), AB (<0.0001), AC (<0.0001), BC (<0.0001), A^2^ (<0.0001), B^2^ (<0.0001) and C^2^ (<0.0001) have significance on the TFC in the obtained extract. Temperature and ethanol concentration, according to the regression Equation (9), show a negative influence on the ethanol extraction of flavonoids, while the extraction time has a positive influence. The interaction of extraction time–solvent and time–temperature, respectively, positively influences the extraction of these compounds from plant material, but the interaction of temperature–solvent shows a negative effect on TFC, according to Equation (9).

TFC = 15.00 − 0.1712A + 1.31B − 0.2468C + 0.6952AB − 1.10AC + 1.31BC − 1.24A^2^ − 1.40B^2^ − 0.5759C^2^ (9)

As can be seen in Figure 1(C1–C3), the interaction between solvent (C) and extraction time (B) exhibited the greatest positive impact, while the interaction between temperature (A) and solvent (C) showed the greatest negative influence on TFC extraction from RGP in the case of conventional extraction. Similarly, the negative impact of the interaction between temperature and solvent on flavonoid extraction from grape pomace was highlighted by Moutinho et al. [19]. However, in their study, the regression equation showed that temperature, solvent and their quadratic terms had a positive effect on TFC, in contrast with our study, where, according to Equation (9) these parameters had a negative impact on TFC extraction from PGR. This negative effect may be explained by a higher concentration of ethanol or temperature, leading to the degradation of flavonoids.

The results obtained for TFC suggest that ethanol has a similar potential in solubilizing flavonoids, but may present a slight difference in efficiency compared to NaDES depending on the extraction parameters. Craciunescu et al. [34] reported a total flavonoid content of 17.16 mg CE/g DW for grape pomace. An equal TFC was reported by Serea et al. [41] in the case of extractions of Băbească Neagră grape skins with 96% and 70% ethanol, acidified with citric acid, 11.34 mg CE/g DW. This variation was strongly influenced by the interaction between extraction time and the type of solvent used. A longer extraction time may allow for a more complete extraction of flavonoids, but the maximum efficiency also depends on the characteristics of the solvent, such as its polarity and its ability to interact with the plant matrix [39].

In the case of the response variable, TFC, for the NaDES extract, all model parameters show significance on this variable, due to its *p*-value less than 0.05. According to the regression equation generated for TFC, the extraction time (B) showed the highest positive influence on the flavonoid extraction from PGR, followed by the quadratic solvent volume, the interaction between temperature and extraction time (AB) and temperature (A). Solvent volume (C) showed the highest negative influence on the response variable, followed by the interaction of extraction time and solvent volume (BC), quadratic of extraction time (B^2^) and quadratic of temperature (A^2^).

TFC = 8.95 + 0.6980A + 1.07B − 3.20C + 0.7775AB − 0.3250AC − 1.27BC − 0.3680A^2^ − 0.4228B^2^ + 0.8942C^2^ (10)

Analyzing the interactions of the studied independent variables on TFC in Figure 2(C1–C3) by means of 3D response surface and 2D contour plots generated based on the results obtained from the polynomial regression Equation (10), it was observed that the interaction of solvent volume and extraction time most significantly influences the extraction of flavonoids from RGP compared to the S/L–temperature and temperature–extraction time interaction, respectively.

The flavonoid content showed a significant variation depending on the solvent used and the extraction time. Dabetic et al. [42] optimized the extraction of phenolic compounds from red grape seeds using an acidified hydroalcoholic solvent (ethanol–water–acetic acid in a ratio of 70:29.8:0.2 *v*/*v*/*v*/*v*) and a NaDES solvent obtained from choline chloride and citric acid in a 2:1 molar ratio with the addition of 30% water (*v*/*v*) in ultrasound-assisted extraction. The authors reported a higher values for TFC, ranging from 44.1 to 102.6 mg CE/g DW. These differences in results may be attributed to the different raw material used for the extraction, which depend on geographical origin, variety, etc.

### 3.5. Influence of Independent Variables on DPPH

Extracts obtained with ethanol were studied in vitro for antioxidant activity using the method presented by Serea et al. [23], and the value of this dependent variable ranged from 5.66 ± 0.007 to 14.25 ± 0.012 mMol Trolox/g DW. ANOVA was utilized in establishing the statistical parameters for the quadratic model; thus, with a confidence interval, the terms within the experimental model that showed significance on the outcome variable (DPPH) were B (<0.0001), AB (<0.0001), AC (0.0002), BC (0.0005), A^2^ (<0.0001), B^2^ (<0.0001) and C^2^ (<0.0001), while C (0.5344) has no significance.

DPPH = 14.22 − 0.0167A − 0.0811B − 0.0079C − 0.1425AB + 0.09AC − 0.08BC − 2.91A^2^ − 2.94B^2^ − 2.49C^2^ (11)

The individual independent variables showed a negative influence on DPPH as a result of the value of the coefficients within the generated second order polynomial Equation (11). The interaction between temperature and solvent showed a positive effect. Anghel et al. [43] claimed that in the case of ethanol extraction, antioxidant activity is influenced by the interaction between temperature and extraction time; thus, for the extraction of grape pomace purees with 4.5 mL 70% ethanol and 0.5 mL acetic acid, they obtained values between 17.09 and 20.07 mMol Trolox/g DW. Serea et al. [41] obtained different values for antioxidant activity, ranging from 3.70 mMol Trolox/g DW, for the 70% ethanol, which was 99.5% citric acid extraction, and 18.76 mMol Trolox/g DW in the case of extraction with 96% ethanol–glacial acetic acid.

An evaluation of the antioxidant activity for extracts obtained from the skins of Băbească Neagră and Fetească Neagră of 3.06 and 4.89 μg Trolox/g DW, respectively, was presented by [44].

The antioxidant activity of extract obtained with NaDES ranged from 5.38 ± 0.26 to 7.24 ± 0.01 mMol Trolox/g DW. The model terms that showed a *p*-value less than 0.05 in the case of antioxidant activity of the extract obtained with NaDES solvent were A (<0.0001), B (<0.0001), C (<0.0001), AB (0.0185), AC (<0.0001), A^2^ (<0.0001) and B^2^ (<0.0001). According to the coefficients in the regression equation (Equation (10)), temperature and extraction time, respectively, positively influenced the antioxidant activity, while the amount of solvent showed the opposite. In the case of temperature–solvent volume (AC), extraction time–solvent volume (BC) factors interaction positively influenced the antioxidant activity, respectively, while temperature–time (AB) interaction negatively influenced it.

DPPH = 6.28 + 0.2170A + 0.1952B − 0.4925C − 0.0263AB + 0.1537AC + 0.0112BC + 0.1314A^2^ + 0.0766B^2^ + 0.0145C^2^ (12)

According to Figure 2(D3) the interaction between temperature and S/L ratio most positively influenced the antioxidant activity of the NaDES extract. Therefore, the antioxidant activity increased directly proportionally with increasing temperature and extraction time. The highest antioxidant activity exhibited a value of 7.24 ± 0.01 mMol Trolox/g DW obtained at a temperature of 60 °C at an extraction time of 60 min.

Both temperature and S/L ratio exerts a significant influence on the antioxidant activity. Values ranging from 18.32 to 57.48 µM TE/g DP and 93.17–140.39 µM TE/g DP were reported by Huamán-Castilla et al. [40]. Sik et al. [45] reported a value for antioxidant activity 30.3 ± 0.18 mg AAE/g when using a glucose–glycerin ratio of 1:1 based on NaDES containing 50% (*v*/*v*) when extracting at a temperature of 25 °C for 120 min.

### 3.6. Optimization and Validation of Extraction Parameters

The desirability value is important for model optimization in the response surface method, as it indicates the best combination of independent variable levels to maximize the desired response (Table 4 and Table 5).

**Figure 3:**
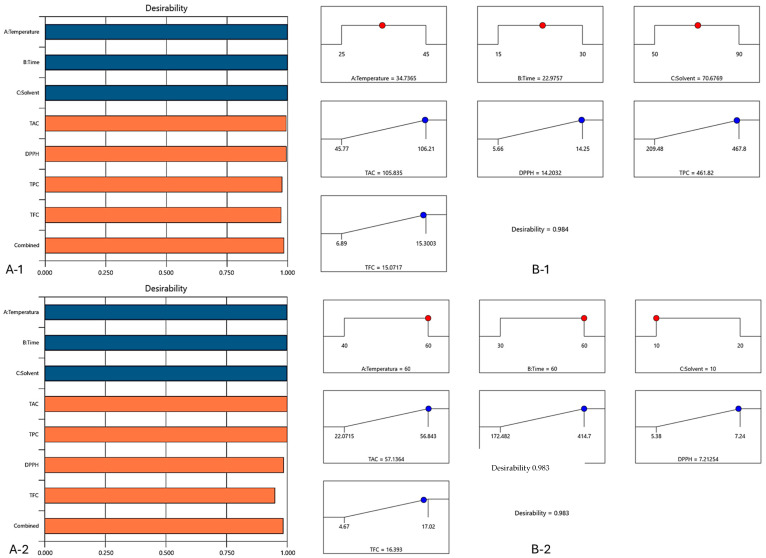
Bar (**A**) and ramp (**B**) plot views of the optimal solutions for the ethanol extract (**1**) and NaDES (**2**) extract. In blue are represented the independent variables, whereas the responses are given in red.

Figure 3 shows the bar graphs and ramps for the two solutions suggested by the program. The bar graphs show the values of the factors’ desirability, describing their individual and combined effects on the responses, respectively, with the main purpose being to obtain desirability values as close as possible to 1. By means of ramp graphs, the main goal is to maximize the desirability, by reaching the steepest possible slope for each dependent variable [23]. The steepest slope obtained for each response highlights the values of the independent variables that lead to the maximization of the dependent variables studied.

Analyzing Figure 3, it can be observed that the values of desirability function for the combination of all variables were 0.984 for ethanol extraction and 0.983 for NaDES extraction; thus, the selected conditions were true. For maximizing the dependent variables (TAC, TPC, TFC and DPPH), the factor parameter values for conventional extraction were 70% ethanol at 35 °C and for 22.5 min extraction duration, while for NaDES extraction were 60 °C for 60 min of extraction and 1:10 mL of S/L ratio.

In Table 4 and Table 5 are presented the predicted values of the limits of variation for the suggested optimal solution in order to maximize the dependent variables of the ethanol and NaDES extracts, respectively. Following the application of the experimental conditions given by the model, for ethanol extraction, the experimental values obtained for the four responses were the following: 105.32 mg C3G/g DW for TAC, 465.81 mg GAE/100 g DW for TPC, 14.15 mMol Trolox/g DW for DPPH, respectively, and 15.3 mg CE/100 g DW for TFC.

Moutinho et al. [19] used a Box–Behnken design by RSM and optimized the extraction parameters (ethanol concentration varying between 55.1% and 72.1%, pH adjusted with 3.5% HCl at 60 °C for 30 min) of grape pomace to obtain the highest yields of the TPC (44.93 mg GAE/g DW), TFC (22.95 mg CE/g DW) and DPPH (0.43 mMol TEAC/g DW).

For NaDES extraction, the experimental values obtained for TAC, TPC, DPPH and TFC following the suggested conditions were 57.58 mg C3G/g DW, 414.04 mg GAE/100 g DW, 7.28 mMol Trolox/g DW and 15.8 mg CE/100 g DW, respectively. The experimental values obtained after applying the predicted independent variables and inputting them into the software were within the given limits of variation, thus demonstrating the validation of the model for the two extracts.

Li et al. [21] optimized ultrasound-assisted NaDES extraction by applying an orthogonal (L_9_ (3^3^)) experiment based on maximizing the yield of TAC from GP. The highest extraction yield of TAC was 5.73 ± 0.05 mg/g under the following optimal extraction conditions: 60 °C, 60 min and a moisture content 25%. From Table 4 and Table 5, it can be observed that NaDES solvent allowed the extraction of a lower amount of TAC and comparable contents for TPC and TFC. The lower concentration of TAC led to a lower DPPH value, indicating a direct correlation between the anthocyanins content and antioxidant activity.

### 3.7. HPLC Analysis of the Extracts

**Figure 4:**
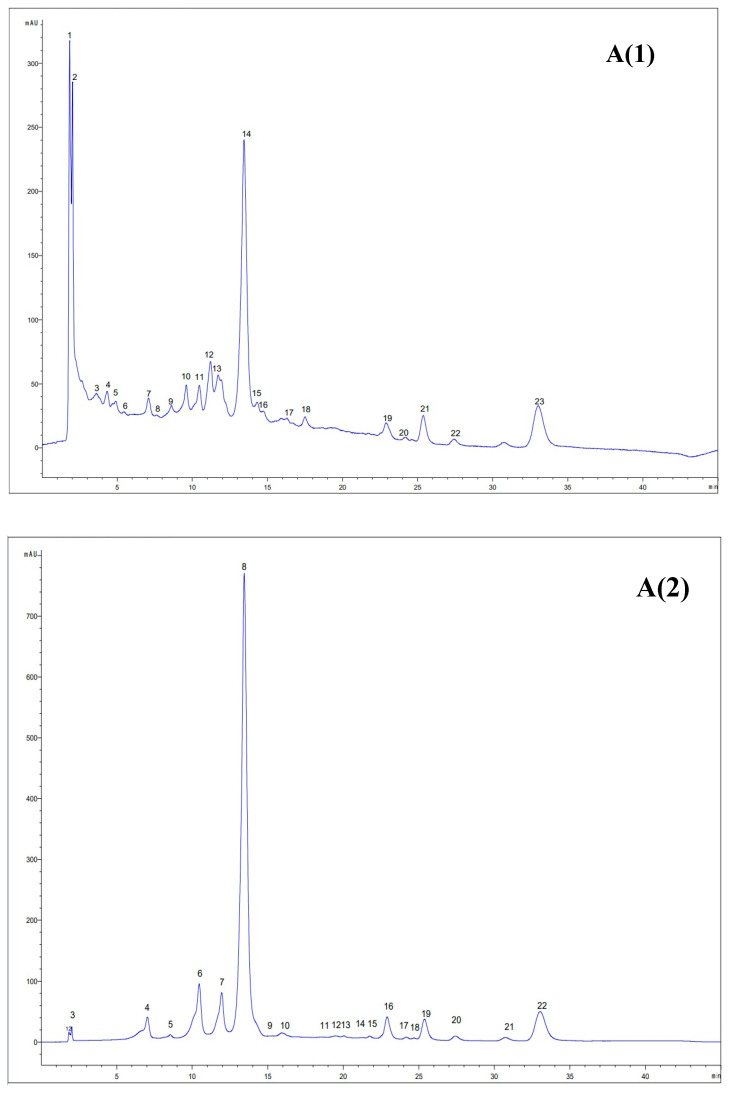

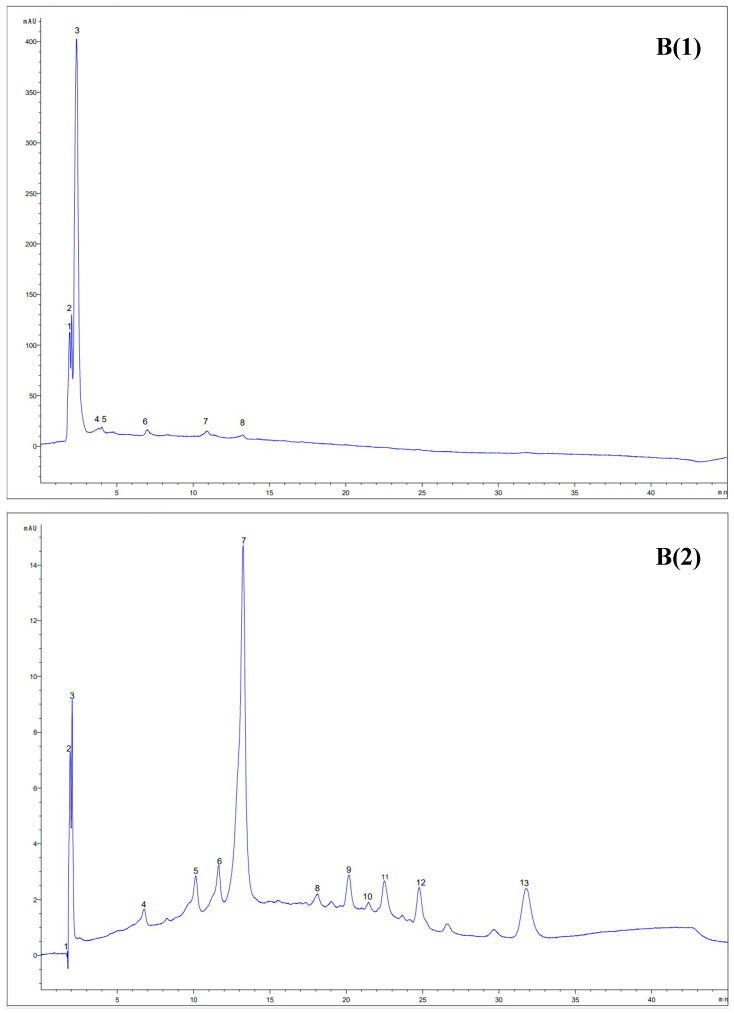
HPLC profile of bioactive compounds of ethanolic extract (**A**) and NaDES extract (**B**): **A**(**1**) 280 nm: 1—unidentified compound, 2—epigallocatechin, 3—unidentified compound, 4—vanillic acid, 5—epicatechin, 6—syringic acid, 7–10—unidentified compounds, 11—keracyanin, 12—unidentified compound, 13—quercetin 3-diglucoside, 14—rutin trihydrate, 15—hesperidin, 16—unidentified compound, 17—cinnamic acid, 18—unidentified compound, 19—kaempferol, 20—unidentified compound, 21—apigenin, 22—isorhamnetin, 23—unidentified compound. **A**(**2**) 520 nm: 1—unidentified compound, 2—gallic acid, 3—epigallocatechin, 4–5—unidentified compounds, 6—keracyanin, 7—unidentified compound, 8—rutin trihydrate, 9–15—unidentified compounds, 16—kaempferol, 17–18—unidentified compounds, 19—apigenin, 20–22—unidentified compounds. **B**(**1**) 280 nm: 1—gallic acid, 2—epigallocatechin, 3—unidentified compound, 4—chlorogenic acid, 5–7—unidentified compounds, 8—rutin trihydrate. **B**(**2**) 520 nm: 1—cafestol, 2—gallic acid, 3—epigallocatechin, 4–6—unidentified compounds, 7—rutin trihydrate, 8–13—unidentified compounds.

The nature of the solvent and the ability to extract compounds with different polarities are important factors in highlighting the bioactivity of natural extracts [46]. The two optimized and validated extracts were subjected to HPLC analysis to establish the profile of bioactive compounds. Therefore, in case of UAE with ethanol, 15 bioactive compounds were identified, among which were 4 polyphenolic acids (vanillic acid, gallic acid, syringic acid and cinnamic acid), 8 flavonoids (epigallocatechin, epicatechin, quercetin 3-diglucozide, rutin trihydrate, hesperidin, kempherol, apigenin and isorhamnetin) and 1 anthocyanin (keracyanin). This contrasts with the eutectic solvent, where only nine bioactive compounds were determined, including five phenolic acids (gallic acid, caffeic acid, protocatechuic acid, ellargic acid and chlorogenic acid), three flavonoids (epigallocatechin, catechin and rutin trihydrate), and also a terpenoid (cafestol) (Figure 4). Epigallocatechin showed the highest concentration in the ethanol extract, 20.57± 2.06 mg/mL, followed by rutin trihydrate, with a value of 1.20 ± 0.04 mg/mL. Similarly to the ethanol extract, epigallocatechin and catechin were the main compounds with the highest concentration in the NaDES extract, with values of 91.77 ± 0.02 mg/mL and 4.51 ± 0.01 mg/mL, respectively.

The differences between the chromatographic profiles can be attributed to the solvents used in the extraction, as well as to the experimental conditions specific to the UAE process, such as temperature and extraction time (Table 6).

The effectiveness of UAE with ethanol has been demonstrated, especially through its high affinity towards compounds from the polyphenol class [46]. However, in a study conducted by Nguyen et al. [47] it was demonstrated that the DES solvent (ChCl:Gly:CA; 1:1:1, *w*/*w*/*w*) was more efficient for the extraction of polyphenolic compounds.

Moutinho et al. [19] extracted polyphenols from the variety “Touriga Nacional”, and “Sousão” extracts were obtained at 60 °C using 3.5% HCl and fourteen 70% ethanol compounds, with different concentrations and with higher concentrations in 3-*O*-caffeoylquinic acid (1.082 ± 0.012 mg/mL), quercetin-3-*O*-rutinoside (0.377 ± 0.002 mg/mL), myricetin-*O*-rutinoside (0.203 ± 0.002 mg/mL). Compared with our study, these authors used a more advanced chromatographic technique (HPLC–DAD–ESI–MS/MS), which allowed for the more efficient separation and quantification of bioactives. The common identified compounds were quercetin 3-diglucoside, kaempferol, apigenin, whereas four anthocyanins were found in their study.

### 3.8. In Vitro Digestibility of Grape Pomace Extracts

By simulating the in vitro digestion of the human gastrointestinal tract for the two optimized extracts, the aim was to generate the necessary information regarding the bioaccessibility of these phenolic compounds during digestion, as well as to determine the amount of TAC, TPC, TFC and DPPH remaining after the completion of the digestion process. The application of this study provides insights into the behavior of these compounds, including their stability and durability, as well as their interactions with enzymes and bile salts within the two simulated environments (SGF and SIF).

The in vitro digestion behavior of bioactive compounds are discussed in terms of the bioaccessibility of bioactive compounds in the gastric stage and intestinal environment, respectively. It has been observed that, for the ethanolic extract, a rapid decrease in TAC was observed in SGF, with an 80% decrease after 120 min of digestion, whereas the NaDES extract showed a good stability, with a decrease of about 10% (Table 7). Therefore, the ethanolic extract showed a lower bioaccessibility, of approximately 21%, when compared with NaDES extract (~91%). The low TAC degradation rate for the NaDES extract can be explained by the chemical composition of the solvent and the pH value provided by lactic acid, which is close to that of simulated gastric fluid [48]. In case of SIF behavior, both extracts displayed a similar trend, with a decrease of 85% and 92% for TAC in ethanolic extract and NaDES, respectively. Therefore, a higher bioaccessibility of TAC in SIF was observed for ethanolic extract of 14.75% when compared with NaDES extract of 7.60%. A similar value of the bioaccessibility index for TAC in the hydroalcoholic extract (70% ethanol extract) was reported by Ferreira-Santos et al. [49] who recorded a value of 17% for the hydroalcoholic extract (ethanol 50%). They highlighted that anthocyanins remain stable in SGF, whereas in SIF, their content decreases drastically due to pH changes and the destruction of the anthocyanin chromophore by enzymes hydrolysis in SIF.

In the case of TPC, the NaDES extract showed a higher stability in SGF, with a decrease of 3%, while in ethanolic extract, the TPC decrease reached 14%. The bioaccessibility showed a high value for both extracts, of about 86% and 97% in the ethanolic and NaDES extracts, respectively. This phenomenon can be attributed to the different pH of the two solvents. The behavior of the hydroalcoholic extract and the NaDES extract after 30 min of gastric digestion showed a slight increase from the initial value of 456.6 mg GAE/100 g DW to 459.95 mg GAE/100 g DW for the ethanol extract, and for the NaDES extract, an increase from the initial value of 473.85 mg GAE/100 g DW to 479.12 mg GAE/100 g DW was observed. After 30 min of SGF, TPC values showed a decrease due to the instability of low molecular weight compounds resulting from the hydrolysis of macromolecular compounds. Also, this decrease in TPC was observed by Kashyap et al. [50] through a three-step in vitro gastrointestinal digestion (oral, gastric and intestinal) study of Meghayalan cherry pomace extracts from the *Prunus nepalensis* variety, obtained by conventional solvent extraction (60% aqueous ethanol, stirred at 50 °C for 12 h) and optimized microwave-assisted extraction (49% aqueous ethanol, 22 mL/g solvent–solid ratio, 450 W power and 197 s extraction time). They observed that the TPC bioaccessibility index during gastric digestion decreased to 52.64% for the microwave-assisted extract and 54.02% for the conventionally extracted sample. In our study, the TPC decreased significantly in SIF for both extracts, with 67% and approximately 75% for ethanolic and NaDES extracts, respectively. Therefore, a lower bioaccessibility was calculated, of 32% and 25%, respectively, with the lower value for the NaDES extract. The SIF behavior is attributed to the pH of the intestinal juice (pH = 7), which alters the ionization state of molecules and affects the stability of phenolic compounds in both extracts [51]. Martinović et al. [52] obtained a hydroalcoholic extract (50% ethanol) using a water bath with stirring at 200 rpm at 80 °C for 120 min. This extract was used as a control in the evaluation of the in vitro bioaccessibility of TPC and TFC for extracts encapsulated through ionic gelation. Compared to the current study, these authors reported a significantly lower TPC bioaccessibility for the control, with a value of 21.25% compared to the optimized hydroalcoholic extract (70% ethanol), with an index for bioaccessibility after SIF of 32.91%, and of 25.16% for the NaDES extract.

Moreover, a higher bioaccessibility of TFC was found in SGS for ethanolic extract of about 89% when compared with 50.56% for NaDES extract, whereas in SIF, the bioaccessibility drastically decreased for both extracts, with values of about 17% and 5% for ethanolic and NaDES extracts.

Regarding the bioaccessibility of the antioxidant activity of the two extracts, a similar value was observed after the gastric digestion. This decrease in antioxidant activity was directly proportional to the decrease in TPC value. The initial DPPH value was 22.63 mMol Trolox/g DW for the hydroalcoholic extract and 23.33 mMol Trolox/g DW for the NaDES extract, while these values decreased to 15.21 mMol Trolox/g DW and 15.69 mMol Trolox/g DW after SGF. Kashyap et al. [50] explained this correlation between the decrease in DPPH value and TPC value as a result of the reduced concentration of antioxidant phenolic compounds during gastrointestinal digestion, following their degradation and hydrolysis.

## 4. Conclusions

In this study, the ultrasound-assisted extraction method was applied to obtain bioactive-enriched extracts from red grape pomace, using two solvents, namely ethanol and NaDES solvent. The optimization using CCD and RSM allowed the testing of different values of the independent variables necessary to achieve the maximum yield of the responses (anthocyanins, polyphenols, flavonoids and antioxidant activity). Therefore, the optimum parameters were established as 70% ethanol, 35 °C and 22.5 min for conventional extraction, whereas for the green extraction, the parameters were 60 °C, 60 min and a 10 mL S/L ratio. Significant different chromatographic profile was evidenced for the two extracts, with an enriched profile in the ethanolic extract compared with the NaDES extract. The optimized and validated extracts were subsequently used for in vitro gastrointestinal digestibility. In general, the polyphenolic compounds extracted with ethanolic solvent showed superior stability during simulated gastrointestinal digestion compared with the NaDES solvent. The obtained results suggest that TAC are more stable in NaDES extract in SGF, as expected, due to the stabilization effect of the pH, whereas the polyphenols and flavonoids showed higher bioaccessibility in the ethanolic extract. The results highlight that even though the temperature and the extraction time for green extraction were significantly higher when compared with the conventional method, green extraction is still a preferred approach when process parameters are not favorable. The extraction yields were similar for total polyphenols, flavonoids and antioxidant activity, except for anthocyanins. However, the use of green extraction is primarily due to its alignment with sustainability goals and its ability to operate without toxic solvents, making it suitable for extracting bioactive compounds from sensitive matrices. When conventional solvents or conditions risk degrading, the target compounds are unsuitable due to environmental or safety concerns, and green extraction provides a viable, eco-friendly alternative. Currently, in further studies, the extracts will be analyzed using advanced chromatography techniques, which will allow a qualitative comparative analysis, whereas in the near future, the extraction with NaDES solvents will continue by testing other solvent combinations, either based on choline chloride and acids or on a neutral basis using polyalcohols.

## Figures and Tables

**Table 1:** Experimental design generated using CCD with dependent and independent variables studied for optimizing conventional extractions (ethanol/water, *v*/*v*).

Run	A: Temperature, °C	B: Time, min	C: Solvent, %	TAC, mg C3G/g DW	TPC, mg GAE/100 g DW	TFC, mg CE/100 g DW	DPPH, mMol Trolox/g DW
1	18.18	22.5	70	65.78 ± 2.77	287.02 ± 0.97	11.59 ± 0.0	6.02 ± 0.005
2	35	22.5	36.36	45.77 ± 1.09	257.04 ± 0.87	13.50 ± 0.29	7.16 ± 0.001
3	35	35.11	70	59.78 ± 0.68	301.05 ± 1.46	13.45 ± 0.0	5.74 ± 0.003
4	25	30	50	46.55 ± 1.35	226.94 ± 0.32	10.49 ± 0.0	6.13 ± 0.003
5	25	15	90	64.13 ± 1.88	256.88 ± 0.75	10.95 ± 0.41	5.81 ± 0.003
6	35	22.5	103.64	61.1 ± 2.10	265.91 ± 0.79	13.20 ± 0.14	7.12 ± 0.015
7	35	22.5	70	105.85 ± 1.04	467.8 ± 1.00	15.30 ± 0.53	14.25 ± 0.067
8	35	22.5	70	105.25 ± 3.86	460.48 ± 0.61	14.93 ± 0.73	14.23 ± 0.042
9	45	30	90	58.34 ± 1.51	303.33 ± 1.61	13.3 ± 0.00	5.66 ± 0.007
10	25	15	50	60.36 ± 1.32	300.14 ± 0.65	12.09 ± 0.18	5.89 ± 0.007
11	51.82	22.5	70	57.73 ± 3.5	300.3 ± 2.60	11.36 ± 0.33	5.92 ± 0.006
12	35	22.5	70	106.12 ± 0.68	460.35 ± 1.06	14.88 ± 0.22	14.24 ± 0.030
13	35	22.5	70	106 ± 2.04	459.82 ± 0.46	14.92 ± 0.32	14.21 ± 0.057
14	45	15	50	58.01 ± 2.77	302 ± 2.48	12.4 ± 0.05	5.93 ± 0.006
15	45	15	90	58.01 ± 2.77	209.48 ± 0.09	6.89 ± 0.36	6.31 ± 0.003
16	35	9.887	70	69.14 ± 1.33	293.33 ± 0.56	8.60 ± 0.00	6.03 ± 0.005
17	35	22.5	70	106.21 ± 2.50	461.87 ± 1.41	15.23 ± 0.37	14.18 ± 0.012
18	35	22.5	70	106 ± 2.04	460.92 ± 0.68	14.73 ± 0.33	14.25 ± 0.012
19	45	30	50	46.45 ± 1.59	274.53 ± 1.65	13.63 ± 0.34	5.7 ± 0.009
20	25	30	90	64.18 ± 1.55	312.00 ± 2.10	14.63± 0.27	5.83 ± 0.003

**Table 2:** Experimental design generated using CCD with dependent and independent variables studied for optimizing green extractions (choline chloride–lactic acid–water 1:2:1.).

Run	A: Temperature, °C	B: Time min	C: Solvent, %	TAC, mg C3G/g DW	TPC, mg GAE/100 g DW	TFC, mg CE/100 g DW	DPPH, mMol Trolox/g DW
1	50	19.77	15	32.26 ± 0.70	184.46 ± 1.69	5.77 ± 0.11	6.17 ± 0.07
2	40	30	10	39.56 ± 8.69	199.80 ± 3.02	9.84 ± 0.37	6.73 ± 0.06
3	50	45	23.41	22.07 ± 0.89	172.48 ± 3.55	5.95 ± 0.58	5.519 ± 0.01
4	50	45	6.59	53.46 ± 1.56	345.90 ± 4.61	17.02 ± 0.00	7.11 ± 0.02
5	40	60	10	46.20 ± 2.88	341.10 ± 9.25	12.66 ± 3.09	7.15 ± 0.01
6	50	70.23	15	45.98 ± 7.31	309.83 ± 1.13	9.75 ± 0.27	6.81 ± 0.92
7	50	45	15	28.19 ± 0.74	197.40 ± 9.98	9.03 ± 0.08	6.3 ± 0.03
8	50	45	15	28.69 ± 0.27	196.30 ± 5.09	9.02 ± 0.03	6.26 ± 0.06
9	66.82	45	15	36.07 ± 4.53	266.20 ± 2.41	9.31 ± 0.22	7 ± 0.05
10	60	60	10	56.84 ± 1.52	414.70 ± 1.40	16.23 ± 0.06	7.24 ± 0.01
11	60	30	10	38.97 ± 0.86	245.40 ± 0.23	9.99 ± 0.11	6.91 ± 0.05
12	50	45	15	29.29 ± 1.58	196.70 ± 5.09	8.65 ± 0.22	6.26 ± 0.02
13	40	30	20	25.58 ± 0.43	197.90 ± 6.87	6.61 ± 0.04	5.38 ± 0.26
14	50	45	15	28.98 ± 2.04	197.20 ± 10.19	9.02 ± 0.08	6.3 ± 0.05
15	50	45	15	28.32 ± 2.76	196.20 ± 4.96	9.11 ± 0.05	6.29 ± 0.06
16	33.18	45	15	26.92 ± 0.81	200.70 ± 6.8	6.52 ± 0.16	6.29 ± 0.03
17	50	45	15	28.00 ± 0.16	196.40 ± 4.84	8.87 ± 0.11	6.25 ± 0.02
18	60	60	20	36.64 ± 0.98	210.10 ± 3.79	6.63 ± 0.19	6.55 ± 0.18
19	40	60	20	26.07 ± 1.64	177.94 ± 2.66	4.67 ± 1.26	5.86 ± 0.07
20	60	30	20	23.35 ± 0.54	202.43 ± 10.38	5.77 ± 0.03	6.19 ± 0.03

**Table 3:** Statistical parameter results calculated by ANOVA for the reduced quadratic model for the dependent variables of the two extracts.

	Ethanol Extract				NaDES Extract			
	TAC, mg C3G/g DW	TPC, mg EAG/100 g DW	TFC, mg CE/100 g DW	DPPH, mMol Trolox/g DW	TAC, mg C3G/g DW	TPC, mg GAE/100 g DW	TFC, mg CE/100 g DW	DPPH, mMol Trolox/g DW
Mean	72.54	333.06	12.80	8.53	34.07	232.46	9.02	6.43
Std. dev.	0.5627	5.18	0.2735	0.0451	0.7039	0.6441	0.2353	0.0264
C.V.%	0.7758	1.56	2.14	0.5288	2.07	0.2771	2.61	0.4112
R^2^	0.9997	0.9983	0.9925	0.9999	0.9974	1.0000	0.9972	0.9986
Adjusted R^2^	0.9994	0.9967	0.9858	0.9999	0.9950	0.9999	0.9947	0.9973
Predicted R^2^	0.9979	0.9885	0.9579	0.9995	0.9838	0.9997	0.9827	0.9924
Adeq. precision	150.6316	67.5342	41.5830	269.0211	69.2065	533.7221	74.1646	96.8130
Lack of fit (*p*-values)	0.0656	0.0503	0.2140	0.0635	0.1269	0.1855	0.1260	0.2754

**Table 4:** Validation of the solution suggested by the mathematical model for conventional extractions (ethanol/water, *v*/*v*).

Dependent Variable	Predicted Value	95% Confidence Intervals	Experimental Value
TAC (mg C3G/g DW)	105.91	104.55–107.26	105.32 ± 0.77
TPC (mg GAE/100 g DW)	461.88	449.41–474.35	465.81 ± 1.28
TFC (mg CE/100 g DW)	15.00	14.34–15.66	15.3 ± 0.30
DPPH (mMol Trolox/g DW)	14.23	14.12–14.33	14.15 ± 0.01

**Table 5:** Validation of the solution suggested by the mathematical model for green extractions (choline chloride–lactic acid–water 1:2:1).

Dependent Variable	Predicted Value	95% Confidence Intervals	Experimental Value
TAC (mg C3G/g DW)	57.14	55.11–59.16	57.58 ± 4.73
TPC (mg GAE/100 g DW)	415.01	431.16–416.87	414.04 ± 0.80
TFC (mg CE/100 g DW)	16.39	15.72–17.07	15.8 ± 2.22
DPPH (mMol Trolox/g DW)	7.21	7.14–7.29	7.28 ± 0.02

**Table 6:** HPLC profile of the extracts.

No.	Identified Compounds	Concentration (μg/mL)	
		Ethanol Extract	NaDES Extract
1.	Cafestol	n.d.	Traces
2.	Gallic acid	n.d.	479.78 ± 84.76
3.	Epigallocatechin	20,576.01 ± 2067.62	4514.44 ± 11.78
4.	Catechin	n.d.	9177.5 ± 21.34
5.	Protocatechuic acid	n.d.	867.88 ± 15.98
6.	Chlorogenic acid	n.d.	227.0797 ± 9.18
7.	Caffeic acid	n.d.	40.80 ± 2.35
8.	Vanillic acid	18.53 ± 0.31	n.d.
9.	Epicatechin	740.51 ± 30.60	n.d.
10.	Syringic acid	5.69 ± 0.08	n.d.
11.	Keracyanin	143.02 ± 4.75	n.d.
12.	Quercetin 3-diglucoside	95.09 ± 1.42	n.d.
13.	Rutin trihydrate (quercetin 3-rutinoside trihydrate)	1204.66 ± 46.34	34.15 ± 1.97
14.	Hesperidin	302.17 ± 19.88	n.d.
15.	Cinnamic acid	6.36 ± 0.22	n.d.
16.	Quercetin	16.80 ± 1.18	n.d.
17.	Quercetin dihydrate	19.59 ± 1.53	n.d.
18.	Luteolin	14.28 ± 0.23	n.d.
19.	Kaempferol	28.70 ± 0.41	n.d.
20.	Apigenin	27.36 ± 0.25	n.d.
21.	Isorhamnetin	11.92 ± 0.11	n.d.
22.	Ellagic acid	n.d.	142.55 ± 8.23

n.d.—not detected.

**Table 7:** Phenolic content during the in vitro digestion.

Extract	Phenolic Content	Before Digestion	After Gastric Simulation	After Intestinal Simulation
Ethanol 70%	TAC, mg C3G/g DW	242.29	50.77	7.49
	TPC, mg GAE/100 g DW	456.6	393.8	129.6
	TFC, mg CE/100 g DW	25.55	22.65	3.77
	DPPH, mMol Trolox/g DW	22.63	15.21	Bdl
NaDES	TAC, mg C3G/g DW	108.77	98.81	7.51
	TPC, mg GAE/100 g DW	473.8	460	115.7
	TFC, mg CE/100 g DW	19.88	10.05	0.54
	DPPH, mMol Trolox/g DW	23.33	15.69	0.17

Bdl = below detection limit.

## Data Availability

Data are contained within the article.
